# Thirty years of climate mitigation: lessons from the 1989 options appraisal for the UK

**DOI:** 10.1007/s12053-021-09951-2

**Published:** 2021-04-02

**Authors:** Eoin Lees, Nick Eyre

**Affiliations:** 1 Eoin Lees Energy, 4 Silver Lane, West Challow, Wantage, Oxon, OX12 9TX UK; 2 Environmental Change Institute, Oxford University Centre for the Environment, South Parks Road, Oxford, OX1 3QY UK

**Keywords:** Climate mitigation, Energy efficiency, Renewable energy, Energy intensity

## Abstract

In April 1989, the UK Prime Minister, Margaret Thatcher, convened a full cabinet meeting on climate change addressed by leading scientists. The presentation on mitigation of carbon dioxide emissions was made by the Head of the Energy Technology Support Unit (ETSU), Ken Currie, and identified the key potential options for mitigation by 2020. In this paper, we compare the mitigation potential identified for each proposed option with the 2019 outturn. The largest mitigation options identified were improved end use energy efficiency across the economy and the generation and use of low carbon electricity. Our analysis finds that these have been the key options adopted. Reductions in primary energy use, resulting from improvements in energy efficiency were concentrated in the period 2005–2012 which in 1989 were widely considered to be ambitious. Decarbonisation of electricity has been achieved by the displacement of coal, initially by gas and more recently by renewable electricity. Renewable electricity has exceeded 1989 expectations in the last 5 years and is now the biggest source of CO_2_ reductions from electricity generation. The contribution envisaged by nuclear electricity has not occurred, largely due its failure to compete in liberalised generation markets. In all cases, the policy environment has been important. We draw lessons for mitigation options to achieve the goal of net zero emissions in the next 30 years. The contribution of demand side and other modular options will remain crucial, as mass-produced technologies tend to improve more quickly than those requiring large construction projects. Environmental, social and political factors will be important, so analysis should not be a purely techno-economic assessment.

## Introduction

The UK was an early mover in understanding the scientific and political importance of climate change. The first significant UK Government political intervention on climate change and its mitigation was in 1989. On 26th April that year, the Prime Minister, Margaret Thatcher convened a cabinet seminar addressed by distinguished scientists (Agar, 2019). The single speaker on climate mitigation was the Head of the Energy Technology Support Unit (ETSU), the late Dr Ken Currie OBE. His presentation and paper were drafted by a small team in ETSU who had assembled the data. Here we use data from the original paper (Currie et al., 1989), which we refer to throughout as the ETSU 1989 report.

The ETSU 1989 report considered options that might be deployed in the UK to reduce carbon dioxide emissions over the period to 2020, i.e. a period of 31 years. It was the first significant attempt to undertake a long-term projection for carbon dioxide emissions from the UK and the scope for their mitigation, predating both the first IPCC report on global emissions (IPCC, 1990) and the early UK climate scenario analyses, by the Royal Commission on Environmental Pollution (RCEP, 2000) and the Cabinet Office (PIU, 2002), that underpinned the UK’s first long-term carbon emissions targets. By modern standards, the data available and analysis were limited, However, the task of considering options for climate abatement over a 30-year period has an obvious resonance, given the current focus on the very ambitious mitigation target for 2050 (CCC, 2019; BEIS, 2019).

This paper takes the opportunity of the 30th anniversary of the ETSU 1989 report to revisit its analysis and conclusions, to compare with actual changes between 1989 and 2019, and to draw out important lessons for the imminent process of 30-year scenario construction.

Decarbonisation implies significant change within energy systems currently dominated by fossil fuels. And energy system transition is inevitably a complex process. A helpful, and increasingly widely used, heuristic is the multi-level transitions approach (Geels, 2002), which emphasises the importance of three levels of activity: innovation (niches), the existing system (regime) and broader societal change (landscape). Forces of continuity and the power of existing actors make systems difficult to change (Unruh, 2000). Change therefore tends to occur when there is pressure on the existing system from both the landscape and niche innovation. National energy policy is therefore an important component of securing change. For this reason, analysts of the energy transition increasingly look to nuanced public policy theories rather than ideas of optimality or rationality, i.e. we are more concerned with how policymaking actually operates rather than abstractions of how it ought to work. In this analysis, we draw particularly on the insight that complex change is likely to require multiple policies (Rosenow et al., 2016). Some of the issues are particularly important on the demand side, where there are multiple barriers to change (Eyre, 1997) and strong evidence of the need for multiple interventions (Brown & Wang, 2015).

So there are well-established theories for thinking about energy system decarbonisation policies. And the literature on future energy and emissions scenarios is vast. However, the focus of this paper is a retrospective analysis of national climate policy. In this field, the literature is comparatively limited. Leach’s early low energy scenario to 2025 was retrospectively reviewed in 1998 (Hammond, 1998), but with respect to primary energy use rather than carbon. And the UK Government’s 2010 target was reassessed in 2001 well in advance of its target date (Eyre, 2001). Whilst there are competing theories of scenario development, there is no agreed methodology for their retrospective analysis. There is only one retrospective meta-analysis of UK energy scenarios (Trutnevyte et al., 2016), which concludes that scenario choices tend to reflect debates at the time of their publication and to emphasise the role of quantifiable variables (e.g. price, growth) over qualitative questions of social and governance change.

This is the first re-evaluation of the outturn of a 30-year national climate mitigation potential study for the UK and to the best of the authors’ knowledge anywhere. Given the novelty of the reassessment and the constraints identified above, our research questions are necessarily modest. They are as follows:

- How do the projections of climate mitigation potential in 2020 contained in the ETSU 1989 report compare with 2020 energy supply and use?
- What are the key reasons for the differences between the mitigation potentials identified and those that have been delivered?
- What lessons can be learned for future 30-year climate mitigation assessments?

The next section of the paper sets out the energy and climate policy context in which the ETSU 1989 report was developed and ‘Background to the 1989 ETSU report’ section provides the background to the report itself. ‘Methodology of the ETSU 1989 report’ section considers the methodology of the ETSU 1989 report. ‘ETSU 1989 appraisal estimates of potential CO_2_ saving’ section then sets out the results of that report. ‘Comparison of ETSU options for CO_2_ savings by 2019 and 2019 outturn’ section compares the mitigation potential identified in the ETSU 1989 report to the 2019 outturn and discusses the key discrepancies. ‘Conclusions’ section draws conclusions and ‘Discussion’ section discusses implications for future projection and scenario exercises.

## The energy and climate policy context in 1989

**Fig. 1:**
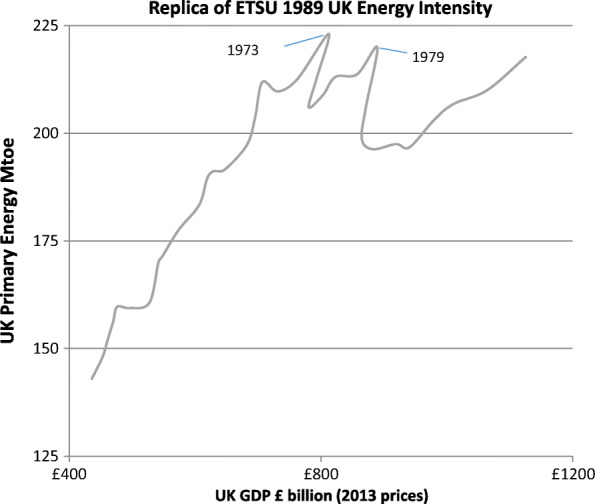
UK energy use and GDP 1954 to 1988. Author calculations based on primary energy consumed and temperature adjusted from the UK Digest of Energy Statistics plus BEIS recent revisions over the period. GDP is in £ billions at 2013 prices (BEIS, 2020a, Table 1.1.4) and primary energy in Mtoe rather than million tonnes of coal equivalent in the original paper

The two major rises in oil price in 1973 and 1979 had caused significant reductions in energy consumption, both by causing recessions and incentivising increased energy efficiency. Although a positive association between energy consumption and GDP was resumed after each oil price rise, the effect was weaker as shown by the decreasing gradient in Fig. 1.

The oil crises of the 1970s marked the beginning of more active government energy policy in much of the world. To counter the power of the OPEC cartel, the major oil consumers formed the International Energy Agency, which has subsequently developed into a leading proponent of low carbon energy. Many Western countries established a specific Department of State and/or national energy agencies to meet the new challenges. In the UK, a separate Department of Energy (DEn) was formed in 1974 and ETSU was established shortly afterwards.

As a result of the oil price rises, from the late 1970s to early 1980s, the received wisdom in energy policy was that future oil prices, and hence energy costs more generally, would be on a steadily upward trajectory. This led to policy support for other fuels, as well as increased attention to energy efficiency. In terms of deployment, the initial focus was on nuclear and coal, but there was also the beginning of a renewable energy R&D programme. The changes observed in energy efficiency in this period were driven by a combination of the general expectation of rising prices and policy support (Mallaburn & Eyre, 2014).

The planning enquiry into the first proposed pressurised water reactor (PWR) in the UK started in January 1983. It was the longest energy enquiry in the UK and was not finally published until December 1986. The received wisdom on future energy prices had a major impact on government thinking and evidence to the enquiry. The Department of Energy produced three scenarios on the likely future costs of electricity and related fuels; these scenarios, created in 1982, all anticipated significant future increases in energy costs.

The Sizewell B enquiry was hotly contested, as energy policy became very politically contentious. The enquiry spanned the period of the 1984–1985 miners’ strike, which lasted 12 months and caused major social divisions. Most attention focussed on the perceived choice between nuclear power, supported by the Conservative Government, and coal, supported by its opponents in the labour movement. But more fundamental, and in the long term more important, other challenges also began to emerge.

Studies were already being produced looking at how to improve energy efficiency (e.g. Lovins, 1977; Leach et al., 1979) and lower the costs of renewable energy (Boyle & Elliott, 1977). More studies followed in subsequent years.

Another important part of the energy policy context was the decision in 1982 by Nigel Lawson (then Secretary of State for Energy) to move to a more strongly market based framework. After the Second World War and until the 1980s, the UK energy industries were nationalised, with the exception of the oil and upstream gas industries. The implications of Lawson’s speech were that these industries would be both privatised and opened to competition, with market forces largely determining prices. The change of policy altered the role of DEn from planning energy provision and supply through state monopolies to one of the Government setting a framework for the energy market to operate (Helm, 2004).

The same deregulatory trends ultimately influenced EU policy, so that in March 1991, the 1975 EU Directive that banned the use of natural gas for power generation was repealed. This stimulated the so-called dash for gas in generation of electricity in Britain in the 1990s (Watson, 1997). This focus on ‘liberalised markets’ tended to limit active support for deployment of both energy efficiency and renewable energy. In both cases, policy focussed on research, development and demonstration (RD&D) and information programmes.

In 1989, the conventional wisdom in UK Government, reflecting the evidence to the Sizewell B enquiry, still considered coal and nuclear power to be the likely contributors to electricity generation until 2010. However, cracks in the argument were increasing. The difficulties that would be faced by nuclear power in attracting private finance were already becoming apparent (Holmes et al., 1987). It was also already known that coal generation would be affected by the growing environmental concerns over acid emissions of NO_x_ and SO_2_, due to the 1988 EU Large Combustion Plant Directive (Skea, 1988; Boehmer-Christiansen & Skea, 1991). In this context, electricity generation from gas was the solution preferred by the market.

It was into this policy context that climate change became an emerging issue. Internationally, there were moves to establish a global governance framework, e.g. from the 1988 Toronto scientific conference on ‘Our Changing Atmosphere’, which called for a 20% reduction in CO_2_ emissions by 2005 and a 50% long-term reduction (UNEP, 1988). As a result, the United Nations agreed to the formation of the IPCC later that year. By 1989, civil society concern about environmental issues was growing with significant votes for green parties in EU elections in the UK and other countries (Curtice, 1989). The IPCC produced its first report in 1990, leading to international agreement to the UNFCCC in 1992.

## Background to the 1989 ETSU report

The Energy Technology Support Unit (ETSU) was established in 1974, following the first oil crisis, to act as the government’s energy agency, by supporting the Department of Energy. It was based at the Harwell Laboratory of the UK Atomic Energy Authority. Early on, a unit known as the Chief Scientist’s Group was established at ETSU to assist the DEn’s Chief Scientist in assessing the technical, economic, environmental and strategic implications of various energy policies or technology strategy options. Additionally, ETSU managed the Department’s RD&D activities concerned with renewable energy sources and energy efficiency; the Buildings Research Establishment (BRE) provided their expertise in in energy efficiency in buildings to ensure in-depth coverage of both key areas.

By the mid-1980s, ETSU was also either involved in, or asked by DEn for opinions on, technical policy issues and responses to external studies, e.g. Leach et al. (1979). Later, it also managed RD&D programmes for DEn on clean coal, fuel cells and developed expertise in using the IEA MARKAL model to assess future UK energy systems, including meeting environmental constraints at least cost.

In 1985, ETSU began one of its periodic assessments of RD&D programmes for DEn. By then it had become clear that the scenarios developed for the Sizewell B Enquiry would not materialise; for example, by 1985 oil prices in real terms had dropped by nearly a half from their 1980 peak price due to Saudi Arabia increasing oil production in order to regain its market share. Initially ETSU was instructed that for their RD&D assessment they should use the scenarios presented to the Sizewell Enquiry in 1982. However as the discrepancy between the Sizewell projections and the real world continued (by 1986, oil price in real terms was less than one-third of its 1980 peak price), ETSU were allowed to introduce a constant real energy prices scenario based on 1985 actual prices to add to the three existing Sizewell B scenarios (ETSU, 1987, Appendix).

The addition of this 1985 constant price scenario to what became UK Energy Paper 54, Energy Technologies for the United Kingdom: 1986 appraisal of research, development and demonstration (HMG, 1987) allowed a more realistic assessment of the cost-effectiveness of the wide range of technologies that had been explored by Government, the energy industry and relevant manufacturers. ETSU thus provided Government with an overview of future energy options.

Even when the Prime Minister, Margaret Thatcher became aware of climate change as an emerging issue in the late 1980s, there was still limited attention to it as an issue in DEn and ETSU, which only began first assessments of basic issues of climate change in 1988. Thereafter, the national and international political pressures changed attitudes rapidly. The Prime Minister spoke to a meeting of the Royal Society on 27th September 1988 and included climate change as one of three environmental issues needing to be addressed by its members. At her instigation, a seminar was arranged on the issue in Downing Street for the Cabinet on 26th April 1989. There were presentations by Professor Tom Wigley, Climatic Research Unit, University of East Anglia on ‘Scientific Assessment of Climate Change and its impacts’ and by Dr Ken Currie, Head of ETSU on ‘Options for Mitigating the Greenhouse Effect’ (Currie et al., 1989). Also present was the UK’s UN Ambassador Sir Crispin Tickell who was a strong advocate of international action on climate change.

The presentation on mitigation was developed quickly in early 1989 by a small team, of which the authors of this paper were part. It drew on the analysis in the 1986 Energy RD&D Appraisal of energy technologies (ETSU, 1987) and ETSU’s practical knowledge from management of the UK programmes on energy efficiency and renewable energy.

## Methodology of the ETSU 1989 report

The methodology of the ETSU 1989 report was of necessity relatively straightforward, as there was limited prior literature on both methods and content of carbon mitigation plans at the national level. The broad approach was to consider individual options already identified within ETSU work and likely to make a significant contribution to the UK energy system by 2020.

The report itself contains no explicit economic analysis, but the economic feasibility of individual options was underpinned by the economic assessment of Energy Paper 54 and its background paper, ETSU R43 (ETSU, 1987). This work classified potential energy technologies into three categories:

- economically attractive: i.e. could make a cost effective contribution to UK energy supply/demand at 1985 real prices,
- promising: could be competitive on predicted technology costs and/or if energy prices were to rise in the future,
- unpromising: on then current expectations, not competitive by 2010 even in at the highest energy prices projected by the Sizewell Enquiry scenarios.

Although 2010 was the date used to classify the technology options, calculations were carried out to 2030 (the cut-off date for the Sizewell projections), but this did not materially affect the conclusions; and with the Sizewell scenarios having rising real energy prices beyond 2010, such prices were judged to be no longer credible by the ETSU 1989 team.

The first steps were to update the ETSU R43 report for any changes in the performance, costs and RD&D results of individual technologies and to update energy price trends. This drew on all ETSU staff and their regular relevant energy industry contacts. Following that, ETSU concluded that there would be no significant contribution to CO_2_ reduction by 2020 from the following technologies:

- nuclear fast reactors
- nuclear fusion
- wave power
- conversion of coal to synthetic natural gas
- conversion of coal to synthetic liquid fuels

In retrospect, all of these judgements were clearly correct.

For those technologies which were deemed likely to contribute to UK emission reduction by 2020, the ETSU 1989 report evaluated total potential by 2020. There was no scenario analysis or assessment of inter-dependency.

Due to the dominance of CO_2_ emissions in global warming, the key role of energy in CO_2_ emissions and ETSU’s particular expertise in energy production and consumption, it was decided to focus exclusively on a reduction of CO_2_ emissions. The first task was to estimate how much ‘business as usual’ carbon dioxide might be emitted by 2020. In turn, this required an estimate of the likely energy intensity of consumption by 2020 and how much GDP might have increased by then. As discussed above, the historic link between rising energy consumption and GDP had been reduced by 1988, but there was still a strong belief in Government that energy consumption and GDP would both increase by 2020 (see Fig. 1).

This forward projection was not a simple task as there was no existing rigorous method for making such projections. Energy demand depends on both energy efficiency and the demand for energy services. The latter is related to economic activity, the structure of the economy and innovation in new energy services. For the business as usual or baseline projection, ETSU took the most recent energy intensity (energy per unit of economic activity) for each sector and fuel and projected the trend forwards assuming the UK’s historic average annual GDP growth rate of 2.25%. There was no consideration of the separate effects of changes in economic structure and technical efficiency, as such methods were not widely available until a few years later (e.g. Howarth et al., 1991; Schipper et al., 1992).

**Fig. 2:**
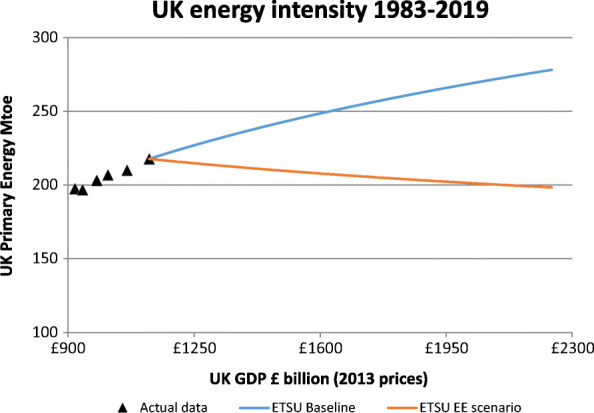
Historical UK energy intensity 1983 to 1988 and ETSU’s business as usual and energy efficiency projection projections to 2020. The primary energy data are for temperature corrected consumption in Mtoe; the GDP data are in £ billion in 2013 prices using the same sources as for Fig. 1

The ETSU 1989 report then developed an energy efficiency projection for the energy intensity that conceivably could be attained by 2020. This drew on the extensive work in ETSU R43 (ETSU, 1987) which had highlighted the enormous potential for reducing the energy required to provide energy services in all end-use sectors. The two projections are shown in Fig. 2. It should be noted that the energy efficiency projection projected a falling demand for energy consumption by 2020.

**Fig. 3:**
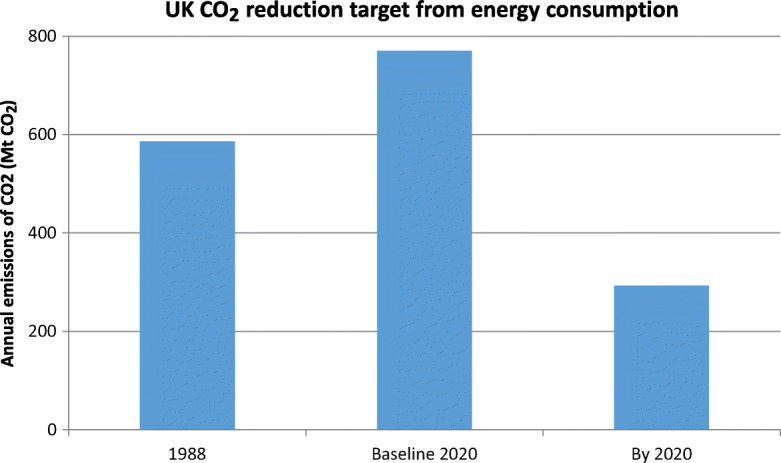
ETSU reduced emissions target for 2020 UK CO_2_ emissions

The next step was to convert the future energy requirement into a carbon dioxide emission figure for 2020. It was assumed that it would be necessary to cut UK emissions by 50% by 2020, from 1988 actual levels, based on the analysis of the 1988 Toronto Conference (UNEP, 1988). As Fig. 3 shows, this required a challenging reduction of 477 Mt CO_2_ (62%) in carbon dioxide emissions from the ETSU business as usual projection assumption for 2020. Equivalently, it required a reduction in annual carbon dioxide emissions to 293 Mt CO_2_.

Options for reducing emissions were then considered individually against this business as usual projection.

We make two presentational changes in units used to account for changes in practice that have occurred in the energy literature since 1989.

- The ETSU 1989 paper measures energy supply and use in tonnes of coal equivalent (tce), in accordance with the dominant practice at the time, with 1 tce defined as 250 therms (26.38 GJ). In our analysis, we follow more recent practice and use tonnes of oil equivalent (toe), defined as 41.868 GJ. The conversion factor is therefore 1.587.
- CO_2_ emissions in the ETSU 1989 report were expressed in tonnes of carbon. Here we have converted these, in accordance with modern practice, into tonnes of carbon dioxide, with a conversion factor of 3.667.

## ETSU 1989 appraisal estimates of potential CO_2_ saving

The eight options identified in the ETSU 1989 report and presented to the Prime Minister and her cabinet are shown in Table 1. Also shown are ETSU’s estimates of the potential savings by each option in terms of the 477 Mt CO_2_ reduction target by 2020. These contributions add to 509 Mt CO_2_ as the individual numbers were derived for each option acting in isolation and inevitably there are complex interactions between the options, which reduce the total when the options are combined. For example, nuclear power, renewables and alternative fossil fuel generation were in competition to replace the then dominant generation by coal.

**Table 1:** The eight options identified in the ETSU 1989 report and their potential contribution by 2020 in million tonnes of carbon dioxide saved

	Option	Mt CO_2_ by 2020	% CO_2_ reduction
1	End use energy efficiency	191	40%
2	Reforestation	11	2%
3	Heat and power from waste	29	6%
4	Transport fuel switching	55	12%
5	Renewable electricity	33	9%
6	CO_2_ capture from electricity generation and sequestration for enhanced oil recovery	10	2%
7	Nuclear power	110	23%
8	Alternative methods of fossil fuel generation	71	15%

The percentage figures in Table 1 are helpful in giving an indication from where the most significant sources of CO_2_ reduction were thought likely to come. The four major opportunities identified were from energy efficiency, nuclear power, alternative fossil fuel generation and transport fuel switching. The rationale behind Table 1 is explained in the rest of this section.

### Energy efficiency

ETSU and BRE had extensively studied improvements in the efficiency of providing energy services in the four main energy-using sectors: residential, non-domestic buildings, industry and transport. ETSU had published, or were working on, four reports addressing the potential of energy saving in each sector to 2000 or 2010 (Langley, 1980; Herring, 1985; Martin & Shock, 1989; Evans & Herring, 1990). These reports identified potential energy savings of 20% savings in industry, 11% in transport, 27% in residential and 36% in electricity end use and 52% fuel saving in the public and commercial sectors. Of particular note were as follows:

- improved insulation in all buildings,
- lighting in all buildings,
- improved building and industrial process controls,
- motors and appliances,
- design of vehicles,
- CHP in industrial and public and commercial sectors

The ETSU 1989 report assumed that all economically attractive energy saving potential would be realised by 2020. Coupled with the assumed business as usual estimate described above, this contribution would mean that energy demand would actually fall by 2020 (as shown in Fig. 2). It would be the largest contributor to the CO_2_ saved by then, saving 191 Mt CO_2_. The business as usual estimate did not precisely distinguish between changes due to economic structure and technical energy efficiency improvement. However, the trends of recent economic restructuring were included in the business as usual projection.

### Reforestation

Based on a study done for ETSU (Mitchell et al., 1987), it was assumed that the 10% of wooded land in UK could be doubled using broadleaved tree species to absorb 11 Mt CO_2_.

### Heat and power from waste

In 1988, waste streams were 2.7 million tonnes of straw burnt, incineration of 2.5 million tonnes of municipal waste, with 28 million tonnes of waste put to landfill. To avoid emissions of methane (with a global warming potential 28 times higher than CO_2_), it was assumed there would be major efforts reduce landfill gas emissions for either electricity generation or combined heat and power. However, the desire by HM Inspectorate of Pollution to reduce landfill emissions and growing concerns over the extent of waste disposal to landfill meant that ETSU expected only a relatively small contribution from straw of 3.5 Mt CO_2_ and the other 25.5 Mt CO_2_ from either the incineration of, or landfill gas collection from municipal solid waste.

### Transport fuel switching

In 1989 transport was the fastest growing end use sector. Road transport accounted for 80% of the transport energy used and within that, cars represented two-thirds of road transport consumption. Clearly alternative fuels for road transport could make a significant impact, but ETSU’s judgement was that electric vehicles and the use of hydrogen as a road fuel were unlikely to contribute much by 2020. The Brazilian experience, dating back to 1976, of steadily increasing ethanol content in petrol, was known, but without the bagasse from sugar cane from which to distil bioethanol, it was judged that in the UK the economics were not promising. Based on optimistic assumptions about increased use of natural gas and liquid petroleum gas (LPG) in vehicles, it was assumed 12% of the CO_2_ target could be met from fuel switching to lower carbon intensity fossil fuels.

### Renewable electricity

In 1988, only hydropower made a significant contribution to renewable electricity, generating 4.8 TWh. Wind energy contributed just 23 GWh and there was negligible electricity generation from solar photovoltaics. The ETSU 1989 report projected that the main new renewable energy contributions to electricity generation by 2020 would come from biofuels, onshore wind, small-scale hydropower, tidal energy and geothermal hot dry rocks.

Using Energy Paper 55 (DEn, 1988), ETSU took the median value of 40 TWh/year from the wide range of electricity production projections by 2020 from these five technologies. Assuming that this additional generation would replace coal, this represented 7% of the CO_2_ savings to be reached by 2020.

### CO_2_ capture from electricity generation and sequestration for enhanced oil recovery

Whilst the idea of carbon capture and storage (CCS) is now familiar, in 1989, it was a radical new proposition. The concept was to remove carbon dioxide from the flues of power stations and then to store it permanently by pumping it down oil wells, which would have the further benefit of enhancing oil recovery from mature wells.

The ETSU 1989 report assumed that by 2020, there would be a 2 GW demonstration coal plant operating at a reduced electrical generation efficiency of 35% and with a 90% CO_2_ capture efficiency, saving 10 Mt CO_2_ annually.

### Nuclear power

In 1988, nuclear power accounted for 20% of electricity production. It was recognised as a low carbon source of electricity and the major source of such electricity at the time. The problems encountered and corresponding delays and increased costs of the advanced gas reactors in the 1960s had led to an abandonment of the plan to build more of these and the Government decided to support PWRs as an alternative. After the Sizewell B enquiry, the UK Government was keen to embark on a significant production line of PWRs. However, given the public reaction to the approval of a Sizewell PWR, it was not clear as to the extent that such a growth plan would be delivered by 2020. For simplicity, the ETSU 1989 report assumed 50% of electricity could come from the existing AGR nuclear plants that would be still open in 2020 and 24 new PWRs. With the increased generation displacing coal, these would save 110 Mt CO_2_ annually by 2020.

### Alternative methods of fossil fuel generation

The ETSU 1989 report considered a range of options to retain fossil fuel generation whilst reducing emissions by substituting gas for coal and/or using more efficient generation, including district heating and combined heat and power. These were as follows:

- substituting natural gas for coal in steam turbines,
- combined cycle power plant using natural gas,
- combined cycle power plant using coal,
- fluidised bed combustion of coal,
- district heating using combined heat and power, and
- fuel cell generation using natural gas.

As discussed above, at the time of the ETSU 1989 report an EU Directive prevented the use of natural gas for electricity generation other than at a small scale. Thus any option using natural gas as a main production fuel was not considered to be significant. In its 1986 appraisal of energy technologies, ETSU had concluded that a 1% improvement in the 37% thermal efficiency of the best existing coal-fired plant was attainable (ETSU, 1987). However, pressure to implement flue gas desulphurisation driven by EU legislation was growing (Skea, 1988) and the CEGB indicated to ETSU that this was likely to reduce the thermal efficiency of the existing coal stations by 1.9% (ETSU, 1987). Consequently, no reduction was envisaged in CO_2_ emissions from the existing coal-fired plants. The contribution from these options was assumed to come largely from alternative coal generation cycles and CHP production, with a contribution of 71 Mt CO_2_.

## Comparison of ETSU options for CO_2_ savings by 2019 and 2019 outturn

In this section, we compare the ETSU 1989 report projections for each option in the 477 Mt CO_2_ reduction target by 2020 with the outturn, based on 2019 UK energy data. It might be argued that we should wait for the 2020 energy data. However, it is already evident that 2020 energy use will be hugely affected by the Covid-19 pandemic, and therefore is unlikely to be a suitable comparator in the context of long-term energy trends.

**Fig. 4:**
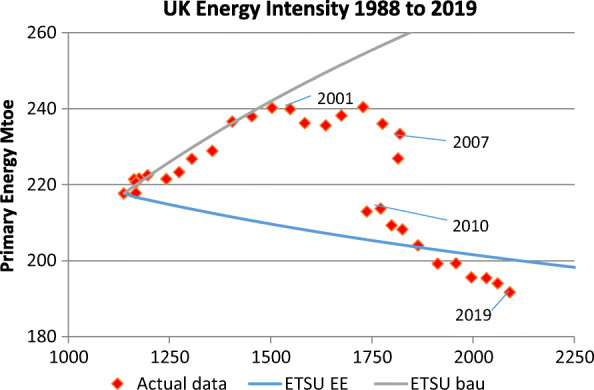
Comparison of 1988 to 2019 of UK energy intensity in the 2 projections by ETSU, energy efficiency (EE) and business as usual (bau), and actual outturn. Authors’ calculation based on official statistics for energy and for GDP using the same sources as for Fig. 2 (BEIS, 2020a).

The data for energy are the most recent Department of Business, Energy and Industrial Strategy (BEIS) statistics (BEIS, 2020a) supplemented by DTI (1992, Tables 47 and 48); for carbon dioxide emissions, we use BEIS provisional UK greenhouse gas emissions (BEIS, 2020b) supplemented by DTI (1992, Appendix C Table C8). Table 2 summarises the relevant figures for 1988 and 2019 as well as the changes between the 2 years and Fig. 4 shows a comparison between the ETSU 1989 report’s projections on energy intensity and the outturn.

**Table 2:** BEIS data on 1988 and 2019 outturns for selected energy and carbon dioxide metrics (sources BEIS, 2020a, BEIS, 2020b and DTI, 1992)

Final energy use	Units	1988	2019	Change from 1988
Primary energy	Mtoe	217.7	191.7	−26
Total final energy use	Mtoe	148.6	142.0	−6.6
Electricity final end use	TWh	274.5	276.8	2.3
CCGT electricity supplied	TWh	Negligible	129.5	129.5
Renewable electricity supplied	TWh	4.8	114.3	109.5
Coal and oil electricity supplied	TWh	225	7.5	−217.5
Nuclear electricity supplied	TWh	55.6	51.0	−4.6
Carbon dioxide emissions				
All energy	Mt CO_2_	590	351.5	−238.5
Electricity generation	Mt CO_2_	197.0	57.4	−139.6
Other energy related	Mt CO_2_	393	294	−99
CO_2_ content of electricity	kgCO_2_/kWh	0.74	0.19	−0.55

There have been significantly more years with low (and even negative) GDP growth than assumed, particularly in the early 1990s and the recession that followed the 2008 global financial crisis. As a result, UK GDP has grown at an average of 1.97% per year rather than the assumed 2.25% per year in the period 1989 to 2019. GDP in 2019 is 8% below the ETSU 1989 report projection and this explains the mismatch of the x-axis in Fig. 4.

Until 2000, energy efficiency improvement was broadly in line with the ETSU business as usual projection so that primary energy final use rose approximately 10%. Energy demand was then fairly flat until 2007, even though GDP grew strongly in early 2000s. Demand since 2007 has declined, most notably during the recession, but the decline continued as economic growth was restored, so that total primary energy demand fell 20% from the 2005 peak to 2019, whilst the economy grew by 21%. In conclusion, total primary energy demand has fallen by 26 Mtoe since 1988. This is a 6.3 Mtoe larger fall than in the ETSU 1989 report energy efficiency projection.

In Table 2, the most notable reductions are in primary energy and the near elimination of coal- and oil-fuelled electricity generation The CO_2_ emission reductions from UK electricity supply and other energy uses are 139.6 Mt CO_2_ and 99 Mt CO_2_, respectively. Allowing for the zero CO_2_ emission generators in 1988 (nuclear and hydro), fossil-fuelled generation (coal and oil were 79% of all generation at that time) had an average carbon emission of 0.88 kg CO_2_/kWh.

By 2019, the primary energy for fossil-fuelled generation had fallen, as coal had been replaced by renewable energy and combined cycle gas turbines (CCGT). In 2019, CCGTs have a thermal efficiency of 48.8% compared to the remaining coal-fired plant of 31.9% (BEIS, 2020a, Table 5.10).

There has also been a dramatic change in the fuel mix of final energy consumption (BEIS, 2020a, Table 1.1.5). In 1988, total energy demand was 148.6 Mtoe and energy demand in 2019 was 142.0 Mtoe on a final energy supplied basis. What is particularly relevant is that the end use of solid fuels and natural gas have reduced since 1988 and only the end use of electricity, petroleum and bioenergy (including waste) have grown. The increase from bioenergy and waste is discussed in ‘Transport fuel switching’ section. Growth in demand for petroleum (due to an increase in transport use) has been slow (62.0 Mtoe in 1988) to 63.1 Mtoe in 2019; if this had been a uniform growth (which it is not), this would have been equivalent to 0.058% per year. Electricity use has risen from 22.8 Mtoe in 1988 to 25.4 Mtoe in 2019; if this had been a uniform growth (which it is not), this would have been equivalent to 0.35% per year. This reflects the growing use of electricity in all end use sectors despite greatly improved energy efficiency as discussed in ‘End use energy efficiency’ section.

The increase in UK renewable energy supplied to the grid has been remarkable and it now contributes 114.3 TWh in 2019, growing from 4.8 TWh in 1988, which was very largely from large hydropower. The nuclear supplied contribution has slightly dropped from 55.6 TWh in 1988 to 51.0 TWh in 2020. The combined addition to UK electricity supply from CCGT and new renewables since 1988 is 239 TWh. This implies that these 2 sources have replaced the lost electricity supplied by coal and oil to the grid of 217.5 TWh.

For UK electricity generation, the rise in gas-fired generation and subsequent growth in renewable electricity has had a distinct benefit for CO_2_ emissions. Compared to 1988 electricity generation emissions of 197 Mt CO_2_, there has been a reduction of 107 Mt CO_2_. This arises from the renewable electricity generation technologies (described in ‘Heat and power from waste’ and ‘Renewable electricity’ sections), the rise in gas-fired generation (‘Alternative methods of fossil fuel generation’ section) and improved energy efficiency in the end services provided by electricity (‘End use energy efficiency’ section).

For the CO_2_ savings from CCGT, we have used the difference between the 1988 fossil fuel figure of 0.88 kg CO_2_/kWh derived at the beginning of this section and the CCGT figure of 0.37 kg CO_2_/kWh (BEIS, 2020a, Table 5E). So the 129.5 TWh supplied (BEIS, 2020a, Table 5.6) has saved 66 Mt CO_2_.

For the CO_2_ savings from new renewables since 1988, we have used the 1988 fossil fuel figure of 0.88 kg CO_2_/kWh derived at the beginning of this section.

For end use energy efficiency, we looked at the actual final energy consumption in 2019 compared to the ETSU business as usual projection, as discussed in ‘End use energy efficiency’ section.

The reduction from actual 1988 emissions required to meet the 50% target reduction for 2020 set out in the ETSU 1989 report would have required a reduction to an annual emission rate of 293 Mt CO_2_; as we are using UK 2019 data, this equates to an annual emission rate of 303 Mt CO_2_. The 2019 actual UK CO_2_ emissions are provisionally 351.5 Mt CO_2_, implying a shortfall of 48.5 Mt CO_2_ from the ETSU 1989 report mitigation target. It should be remembered that the target and its constituent parts were not a prediction, just an analysis of options for policymakers to consider. However, where there are discrepancies between what has been achieved and the mitigation potential identified 30 years ago, it is illuminating to understand why this has happened.

Table 3 summarises the ETSU 1989 report mitigation option assessment and the actual 2019 outturn for UK CO_2_ savings. The outcomes for the individual options are detailed in the rest of this section.

**Table 3:** Summary comparison of ETSU options for CO_2_ savings and reality by end 2019

Option	ETSU 1989 report 2020 mitigation potential (Mt CO_2_)	Mitigation achieved by end 2019 (Mt CO_2_)
End use energy efficiency	191	224
Reforestation	10	10
Heat and power from waste	29	27
Transport fuel switching	55	6
Renewable electricity	33	69
CO_2_ capture from electricity generation	10	0
Nuclear power	110	0
Alternative methods of fossil fuel generation	71	66
Total	509	402

### End use energy efficiency

End use energy efficiency is about providing the same or better energy service whilst reducing the energy consumed. Therefore to get a measure of the CO_2_ savings from end use energy efficiency, we need to focus on final energy use, as some of the primary energy savings will come from CCGT gas-fired generation and renewable electricity. Therefore, we need to evaluate against the final energy demand projections from the ETSU 1989 business as usual projection. This drew on the 1983 to 1988 trends in end use energy as follows: energy demand was assumed to grow annually by 1.2%, 1.6% and 0.5% for transport fuels, electricity and heating fuels, respectively. The different rates of increase were based on past data and reflected expectations about structural changes in the economy away from heating towards transportation and services provided by electricity. In Table 4, we contrast these business as usual projections with the actual outturn.

**Table 4:** Final energy demand projections and outturn by fuel type (author calculations based on BEIS, 2020a Table 1.1.5 and growth rates from the ETSU business as usual (bau) projection).

End use energy demand	UK final energy demand by fuel type (Mtoe/year)				Reduction in demand from bau (%)	CO_2_ reduction (Mt CO_2_)
	1988 actual	ETSU 1989 sector growth rate	ETSU 2019 bau projection	2019 actual		
Electricity	22.8	1.6%	37.3	25.4	32%	123
Transport fuels	45.1	1.2%	65.2	54.5	17%	33
Heating fuels	80.7	0.5%	94.2	62.2	34%	68
Total	148.6		196.7	142.0	28%	224

The largest absolute reductions in energy demand have been in heating fuels a 34% reduction compared to ETSU 1988 bau; this has saved 68 Mt CO_2_. However, electricity efficiency improvements have contributed more to carbon emissions reduction, because of the high emission factor of electricity in 1988; a 32% reduction compared to ETSU 1988 bau; this has saved 123 Mt CO_2_. This is despite the increasing demand for services provided by electricity which have evolved from 1988 in all end use sectors. In the transport sector, a 16.5% reduction in energy demand compared to ETSU 1988 bau has saved 33 Mt CO_2_.

Caution is needed in ascribing these changes solely to energy efficiency technology improvement. As explained in ‘ETSU 1989 appraisal estimates of potential CO_2_ saving’ section, the ETSU 1989 report did not distinguish between changes due to economic structure and technical energy efficiency improvement, and therefore we can only precisely report on the combined effect—the change in energy intensity. For the purposes of this report, we assume that the changes set out in Table 4 represent energy efficiency improvement, and the growth rates assumed in the business as usual projection reflect the structural changes to the economy over the period. This is consistent with what is known about the relative contributions to energy intensity reduction of technical efficiency improvement and structural change (Hardt et al., 2018 who found that offshoring contributed 28% of energy demand reduction from UK productive activities in the period 1997 to 2013).

It is clear that energy efficiency improvement has been a major contributor to emissions reduction across all forms of energy use. Averaged over the period, improvements have been approximately 1% per year. It is also worth remembering the increasing demand for services provided by electricity since 1988 in all end use sectors. For example in the residential sector, entertainment, information and communication, and induction hob cooking are popular innovations. Yet electricity demand in the residential sector in 1988 was 64.7 TWh with 64% used to provide lighting and refrigeration; whereas in 2018, residential electricity demand was 65.8 TWh with only 34% used to provide lighting and refrigeration (BEIS, 2020c).

In summary, the reduction in energy demand has been almost the same as set out in the ETSU 1989 report’s energy efficiency projection, which was widely judged to be ambitious at the time. However, this has not been a smooth transition, with an initial rise followed by recent rapid falls. A number of factors have contributed, including the ‘off-shoring’ of the manufacturing products used in the UK, which was most prominent in the first decade of the 2000s as the Chinese economy grew quickly (Hardt et al., 2018).

Making the assumption that the additional reduction in final energy demand is solely due to energy efficiency, the mitigation due to energy efficiency is 224 Mt CO2, i.e. 33 Mt CO_2_ greater than the 191 Mt CO_2_ projected in the ETSU 1989 report energy efficiency projection. Even taking into account the uncertainty due to allocation between technical efficiency improvement and economic restructuring, energy efficiency is certainly the largest contributor to observed mitigation.

From 2005, the rapid reduction was driven by the stronger energy efficiency policies adopted at both EU level and in UK following 2004 Energy Efficiency Action Plan (Defra, 2004). Key changes were major increases in building energy efficiency (Lees, 2008; Rosenow, 2012) and higher efficiency boilers, vehicles and appliances, all driven by some form of regulatory policy instrument. The key overall finding is the substantial energy efficiency has been achieved, although the policy effectiveness over time has been mixed (Mallaburn & Eyre, 2014). Effective policies have generally been ones that focus on individual sectors or even technologies, recognise the range of barriers that need to be addressed and therefore use a wide range of policy options including regulation, incentives and information.

### Reforestation

Government data on greenhouse gas emissions in 2019 (BEIS, 2020b) show negative emissions (i.e. net absorption) of −11.7 Mt CO_2_ for land use, land use change and forestry. Since 1990, the increase in land use CO_2_ sequestration has been 9.3 Mt CO_2_, so the improvement from 1988 is probably around 10 Mt CO_2_. This is very similar to the mitigation potential of 10 Mt CO_2_ set out in the ETSU 1989 report, although the latter figure was solely for reforestation.

### Heat and power from waste

In 1988, landfill gas and sewage gas provided 0.4 TWh electricity. By 2019, generation from these sources plus anaerobic digestion and energy crops had increased to 31.5 TWh (BEIS, 2020a, Table 5.6). In this analysis we exclude incineration of municipal waste, tyres etc. (5.9 TWh in 2019) as this has not been counted as a renewable energy source in UK Government statistics since 2013. As eligible electricity from waste has substituted for coal-fired electricity with a carbon content of 0.88 kg CO_2_/kWh, the savings have been 27.4 Mt CO_2_.

By 2019, there had been a significant expansion of wood and other energy crops as a heating fuel, so that renewable bioenergy provided 4.0 Mtoe of heat (BEIS, 2020a, Table 6.1) dominated by 2.3 Mtoe wood used in the residential sector and 1.1 Mtoe plant biomass mainly in industry. Reliable data for earlier years on wood burning are not available and the 1988 figure of 0.16 Mtoe is likely to be an underestimate as the Government survey of this energy source (BEIS, 2015) confirmed that historical data had been underestimated by up to a factor of 3. Unfortunately data were only corrected back to 2008 and so the actual figure for all renewable heat in 1988 of 0.32 Mtoe could be as high as 0.6 Mtoe. It is likely that renewable heat has saved between 3.4 and 3.7 Mtoe. CO_2_ emission savings depend on the fuels replaced, which are not precisely known. Assuming oil was the displaced fuel, the savings could be up to 10 Mt CO_2_.

Even allowing for the uncertainty in the CO2 reductions from heat, we are confident that these renewable sources have still managed carbon dioxide savings greater than the potential identified in the ETSU 1989 report, despite combustible municipal waste streams etc. being no longer counted as a renewable resource.

### Transport fuel switching

Despite considerable increases in annual vehicle mileage since 1988, the increase in CO_2_ emissions from road transport has been modest at 5.4 Mt CO_2_. This is primarily due to improved vehicle energy efficiency, including through the increase in more efficient diesel engines, with diesel use rising from 29.75% of road transport fuel in 1988 to 66.9% in 2019. The switch to diesel cars has been much faster than the 1989 ETSU report envisaged. Road vehicle efficiency improvements have been driven by EU requirements for carbon dioxide emissions from cars, although actual performance has been shown to be significantly worse than the test results against which compliance is judged (Anable & Goodwin, 2019) and the recent rapid growth in the market share of sports utility vehicles (SUVs) is also undermining progress (Anable et al., 2019). These improvements in vehicle efficiency are included in the analysis of energy efficiency above.

The use of biofuels in road transport has also contributed to emission reductions, rising from negligible levels in 1988 to 753 million litres of ethanol (1.6% of total road transport fuel) and 1598 million litres of biodiesel (3.4%) in 2018; LPG and natural gas consumption remain negligible at less than 1000 tonnes. Using the BEIS website for UK Government greenhouse gas reporting conversion factors, the CO_2_ savings in 2019 were, for bioethanol 1.65 Mt CO_2_, and for biodiesel 4.04 Mt CO_2_, so a total of 5.7 Mt CO_2_ overall saving from transport biofuels.

The Renewable Transport Fuel Obligation (RTFO) increased the percentage of biofuel production for transport fuel producers from 7.25% in 2018 to 8.5% in 2019. Even with these increases, savings from transport fuel switching will be significantly less than the 55 Mt CO_2_ potential identified in the ETSU 1989 report. The potential identified was primarily from road fuel gases which have not proved an attractive option for vehicle manufacturers despite some UK fiscal incentives. Instead, savings have been delivered through the introduction of biodiesel and the substitution of bioethanol for petrol. The ETSU 1989 report did not anticipate that EU legislation (EU, 2009) would require member states, by 2020, to source 10% of petrol and diesel from biofuels.

### Renewable electricity

The generation of renewable electricity was one area where the potential identified in the ETSU 1989 report significantly underestimated what subsequently has transpired. Even excluding the 31.5 TWh bioenergy electricity generation discussed earlier, 82.8 TWh were generated by renewables in 2019, primarily from wind (onshore and offshore) and solar photovoltaics, compared to the ETSU 1989 report potential estimate of 45 TWh/year by 2020. Renewable generation in 1988 was 4.8 TWh (DTI, 1992, Table 47).

The reason for ETSU’s underestimate was the failure to anticipate the dramatic drop in costs of wind and photovoltaics and the active policy support for their deployment**.** In the last 5 years, the economics and deployment of wind and solar energy have changed far faster than envisaged. Globally, the installed costs of wind turbines have fallen by a factor of three in real terms since 1989 and the costs of large-scale solar PV in the UK have fallen by 77% since just 2010 (IRENA, 2019). At the same time, the UK has been required under the EU, 2009 Renewable Energy Directive to progress towards supplying 15% of total energy demand from renewables by 2020, and this has been cheaper to deliver through electricity generation. In the UK, offshore wind development has been most significant. Contracts for offshore wind generation with the UK Government fell from 15.5 p/kWh in 2015 to 4.0 p/kWh in 2019. The progress with multi-MW PV farms has been similarly impressive, with contracted prices under the GB Feed in Tariff falling from 12.21 p/kWh in 2015 to 5.43 p/kWh in 2019. In both cases, costs are now approximately equal to or less than the wholesale electricity price, which was typically 4.5 p/kWh in 2019 (Ofgem, 2020).

As set out in the introduction to this section, the additional 78.0 TWh of generation achieved in 2019 substitutes for electricity with carbon content of 0.88 kg CO_2_/kWh, resulting in an estimated saving of 68.6 Mt CO_2_.

To a significant extent, the growth in deployment of renewable energy in the UK has been contingent on the price reductions for wind and solar technology that have been a function of global markets. Nevertheless, globally public policy to support deployment has also been critical. The EU Renewable Energy Directive was a key driver, but it still needed to be implemented at a national level. Electricity wholesale market reform that enabled low-risk contracts for investors has been critical, especially for offshore wind.

### CO_2_ capture from electricity generation

There has been no significant use of carbon capture and storage in the UK to date. The International Energy Agency’s programme on CCS research did not begin until 1991 and the first demonstration, for enhanced oil recovery in the Norwegian Sleipner field, was not operational until 1996. Commercial interest in building a demonstration power station with CCS was shown in the period 2010 to 2015 and there were two preferred bidders agreed by the government, SSE and Shell, and the White Rose Consortium. SSE and Shell proposed to capture CO_2_ from a 426 MW coal-fired plant near Drax. However, the government cancelled all grant funding in 2015 and consequently no CCS plant has yet been built in the UK. In summary, the ETSU 1989 report was justified in being cautious about the potential of an untried technology.

### Nuclear power

In reality, no nuclear power plant construction has been completed in UK since Sizewell B was commissioned in February 1995, and none is now expected until 2025 at the earliest.

The ETSU 1989 report assumed that the five existing advanced gas reactors (AGRs) and two then under construction would continue to operate and that there would be a large additional fleet of PWRs constructed by 2020. This was based on Government policy enunciated at the time, and therefore not an unreasonable assumption. Through RD&D, it was believed that the lifetime of the AGR reactors could be increased to 35 years and that their annual load factor could be improved to 74%. The first objective was met, as initially was the load factor improvement. However, the 2019 annual load factor dropped to 62.9% (BEIS, 2020a, Table 5.10) resulting in the energy supplied in 2019 by nuclear power being 51.0 TWh, which represents a decline in nuclear supplied of 4.6 TWh from 1988. Thus the 23% contribution of additional nuclear power to reducing CO_2_ emissions by 2020 has not materialised. In retrospect, this was clearly the biggest single mitigation potential identified in the ETSU 1989 report that has not been delivered.

The reasons for the failure of the UK nuclear programme have been discussed extensively elsewhere (MacKerron, 2004) and will not be repeated here. For the purposes of this paper, what matters is why the outcome and the ex-ante potential are so different. In 1989, the controversial aspects of nuclear power relating to wastes, accidents and weapons were already well-known. In retrospect, the issue that policymakers seriously neglected was the interaction of the proposed nuclear programme with the broader energy policy priority for market liberalisation. Thirty years of experience have indicated that the two policies appear to be inconsistent. Although initial signs were becoming apparent in 1989 (Holmes 1987), the reality was not so clearly established.

### Alternative methods of fossil fuel generation

The ETSU 1989 report envisaged significant contributions to emissions reductions from a range of fossil fuel technologies, including cleaner coal technologies. In practice, fuel switching to natural gas has been the dominant effect, principally to combined cycle gas turbines, CCGT. Gas-fired power stations supplied 129.5 TWh in 2019 (BEIS, 2020a) compared to a negligible amount in 1989.

For the CO_2_ savings from CCGT gas replacing coal-fired generation, we have used the difference between the 1988 fossil fuel figure of 0.88 kg CO_2_/kWh derived at the beginning of this section and the CCGT figure of 0.37 kg CO_2_/kWh in 2019. So the 129.5 TWh of CCGT has saved 66.0 Mt CO_2_.

It is clear that gas has taken a much bigger share of the electricity market than envisaged in the ETSU 1989 report. The critical factor has been the legislation that allowed natural gas to be used as a fuel for electricity generation. The liberalisation of the GB electricity market created new entrants who wanted to build low cost capacity quickly and combined cycle gas turbines were the technology of choice. The combined effect prompted the ‘dash for gas’ in power generation (Watson, 1997). Coupled with increasingly stringent constraints on acid emissions from coal-fired power stations, this has resulted in coal-fired generation coming out of the electricity mix quicker than anticipated in 1989.

## Conclusions

The differences between the mitigation potentials identified in the ETSU 1989 report are summarised in Table 3 and our analysis of the discrepancy between the mitigation potential identified for each of the options is set out in the relevant sub-sections of the previous section. This section aims to draw out some higher level lessons.

Projecting forward expectations of energy production and demand is a difficult exercise. The ETSU 1989 report attempted to project forward 31 years. The expected contributions of different changes to the overall mitigation effort were, arguably, reasonably good. Improvements in energy efficiency have made the largest contribution, ultimately achieving as much as set out in the ETSU 1989 report, although with a different trajectory due to the lag of implementing energy efficiency on a significant scale till 2005.

Low carbon electricity has provided the second largest contribution, although the balance between nuclear and renewable contributions has been very different from what was expected in 1989. We put this down to two factors. Firstly, the commitment in Government to a major new nuclear programme of pressurised water reactors ultimately proved incompatible with the policy commitment to liberalised electricity markets. This potential conflict was known at the time, but the outcome was unpredictable; for example, the UK, like France, could have de-emphasised liberalisation and pushed on with a state-led nuclear programme. Secondly, there has been a rapid rise in the role of renewables for electricity generation in the last 5 years. The ETSU 1989 report’s estimated potential of 45 TWh/year was not exceeded until 2014, but that contribution has now been more than doubled. And the potential of a significant contribution from solar PV was completed missed in 1989.

The successful low carbon policies for energy efficiency and renewable energy adopted by the UK since 1988 have been driven by different policy and regulatory levers, which can be broadly by divided into three categories.

The first category is policies driven by UK Government. These include specific regulatory interventions in buildings, notably the requirement for all replacement boilers to be condensing from 2005, which alone gives a 25% improvement in energy efficiency (Elwell et al., 2015). Early privatisation and re-regulation of the energy sector in the 1990s prompted the UK to be the first country in the world to use energy efficiency obligations (EEOs) on electricity and gas companies in liberalised markets from 1994. These were increased in size over many years up to 2012 (Rosenow, 2012) and influenced the development of similar policies in other countries (Eyre et al., 2009; Bertoldi et al., 2010; Fawcett et al., 2019). The scale of obligations has been reduced substantially in recent years as part of the unsuccessful Green Deal policy package (Rosenow & Eyre, 2013). Some financial support schemes for renewable electricity in the UK also pre-dated EU level drivers (Mitchell, 1995).

The second categories are policies driven by EU Directives but with discretion for the UK to adopt the policy or regulatory mechanisms. In the buildings sector the Energy Performance of Buildings Directive (96/92/EC) has shaped buildings regulations in all parts of the UK and across the EU (Economidou et al., 2020). The Energy Efficiency Directive (2012/27/EU) established an explicit energy saving target for all member states. The Renewables Energy Directive (2009/28/EC) set explicit targets and rules for to member states, including a 15% renewable energy contribution for UK total energy needs, which has proved very influential in driving UK renewable energy policy.

The third category is EU Directives related to traded goods, which have direct EU wide requirements. The Energy Labelling Directive (1994/2/EC) paved the way for differentiation between ‘good’ and ‘bad’ EE products in terms of their energy efficiency. Whilst the initial direct impact on consumer decision making was modest, the label underpinned many of the EEO activities in the UK. The Ecodesign Directive (2009/125/EC) including minimum energy performance standards for energy using products has had a very large impact in securing absolute reductions in appliance electricity use. Similarly, the Vehicle Labelling Directive (1999/94/EC) enabled the introduction of mandatory standards for new vehicle fuel economy from 2015. This has proved effective although it is well-documented that actual fuel economy has not matched test data (Brand, 2016).

Now that the UK has left the EU and the European Single Market, it is clear that in planning for a future net zero UK, more attention will need to be paid to those areas of policy in which EU Directives which have previously shaped progress. This applies particularly in the field of energy using products in which EU policymaking has been central. The UK market is small compared to the EU, and the domination of most markets by multinational manufacturers is likely to make the creation of separate UK product lines uneconomic. Maintaining the undoubted benefits that UK energy efficiency has had from these EU directives points towards retaining close alignment in such standards.

A missing element in the 1989 ETSU report’s analysis was the neglect of political and institutional change. Most importantly, the analysis did not allow for gas-fired generation, for the very straightforward reason that it was not legal at that time under European Law. However, this is not an adequate excuse; laws and governments are just as susceptible to change as technologies, and should be allowed for in foresight exercises. Our analysis shows that changes in legislation, environmental considerations, technical innovation and market mechanisms all play a part in determining climate change policy. For example, the initial decline in coal use was been driven by the eligibility of natural gas as an electricity fuel and by acid emissions legislation; the rise of renewable electricity and biofuels by explicit legal requirements; and energy efficiency improvements by a combination of EU product and vehicle regulations, national building regulations and energy efficiency obligations on energy suppliers.

The obvious conclusions are that a single projection of the future was not a robust approach to foresight, nor the simple assumption that future energy prices are the main determinant of change. Even at the time, foresight with no attention to broader issues was not best practise. Shell had already established a process of scenario planning as early as 1965 (Wilkinson & Kupers, 2014). Such approaches were not widely used within UK Government at the time, although subsequent futures exercises in UK public policy have been far better in this regard. Indeed, the subsequent appraisal of RD&D (ETSU, 1994) followed the scenario planning approach.

By revisiting this work from 30 years ago, we can draw some relevant conclusions for the very much larger body of analysis now being undertaken for the next 30 years of climate mitigation, both in the UK and more widely. There are three important issues that emerge, which we discuss in the next section.

## Discussion

### The central role of energy demand

First, it is important to give a central role to the future of energy demand, not just changes to energy supply. The major success of the ETSU 1989 report was that it correctly identified energy efficiency improvement as likely to be the biggest contributor to climate mitigation over the following 30 years. Major contributions were projected to come from efficiency improvement across the economy and in essence this is what has happened. Although perhaps uncontroversial now, it was viewed with suspicion within government and much of the energy industry at the time. A similarly unjustifiable resistance to recognising the potential for energy efficiency identified in earlier work (Leach et al., 1979) is reported in Hammond (1998). In retrospect, it is clear from Table 3 that the relative importance of energy efficiency was probably slightly underestimated in the ETSU 1989 report and over half of actual mitigation has been due to energy efficiency.

In some ways, little has changed; most international analysis shows that continued demand reduction will be critical (Edenhofer et al., 2014; Grubler et al., 2018). Linked to electrification of many other end uses, energy efficiency and demand side management have the potential to justify being the dominant approach to thinking about decarbonisation. Yet, current UK Government policy set out in the Clean Growth Strategy (BEIS, 2017) and the Energy White Paper (BEIS, 2020e) still presents energy supply as the primary instrument of climate mitigation. Energy efficiency policies have been weakened since the period of their major success from 2005 to 2012, reflecting that some of the key barriers to energy efficiency are political (Mallaburn & Eyre, 2014). Recent policy to address the post-pandemic recovery included stronger support for building energy efficiency, but was still conceived of as a short-term fix rather than part of a long-term energy and climate strategy and has consequently failed to deliver significant change. It seems highly unlikely that decarbonisation at the rate hoped for can be achieved without this decline being reversed.

### The role of small-scale, mass-produced technologies

Secondly, there are lessons about the plausible rates of technological change. Of course, technology assessment 30 years into the future has some irreducible uncertainties, but there are lessons we can learn. In the ETSU 1989 report, the dominant approach to innovation was focussed on what RD&D might achieve to reduce costs. There was little attention to cost reduction in production; this was perhaps not surprising as there was no significant use of the learning curve concept in energy policy until early 2000s (McDonald & Schrattenholzer, 2001).

The lessons of the last 30 years are that cost reductions can be significant and even dramatic when technology is manufactured in very large volumes. Progress in wind and PV technology has exceeded expectation as confirmed by the recent BEIS publication on the continuing falling costs of renewable energy (BEIS, 2020d). There are similar examples in energy demand technology: notably light emitting diodes (LEDs) now dominate sales of new lighting despite being an unproven technology in 1989; and using product regulation to lower the standby electricity consumption of electrical goods and to improve the efficiency of gas boilers. Energy use by information, entertainment and communication technology has been transformed. In these cases, innovation has been successful because technological improvement and social acceptance in early niche applications has been accompanied by new ‘landscape priorities’ driven by concerns about climate change. This combination has enabled change in energy sector companies and key supply chains to adopt new technologies and approaches at scale.

However, such progress in cost reduction is not universal. Much of what was written in 1989 about nuclear fission, fusion, large tidal barrages, wave energy and CCS could be reproduced today without much amendment. All technologies have uncertain cost futures. However, in general, technologies dependent on large engineering projects have demonstrated less significant cost reductions from experience than mass-produced manufactured technologies. Therefore reliance on significant future cost reductions in these areas seems unwise and not justified by our experience to date.

Such lessons are relevant to current debates. There is widespread discussion about negative greenhouse gas emission solutions, in particular using biomass energy carbon capture and storage (BECCS). The driver of this interest is obvious. If we are to achieve ‘net zero’ in a system where some sources of greenhouse gases are difficult to abate, then some negative emissions may be needed. However, BECCS requires large construction projects and back-to-back contracts with environmentally sensitive fuel and waste disposal developments (Gough et al., 2018). This is the type of innovation which would be expected to deliver low rates of learning and social acceptance.

### The role of public policy

Thirdly, progress is not just a matter of technical change. The adoption of legal and regulatory measures has been critical to delivering progress in UK climate mitigation. Progress has been most rapid in periods when regulatory measures were used (Mallaburn & Eyre, 2014) and the reluctance to use such measures has been responsible for the biggest setbacks (Rosenow & Eyre, 2016). In some cases, this has been achieved via highly visible and popular measures such as the PV feed in tariff, which enabled solar generation to develop as a large niche market on one million buildings and in community energy initiatives (Smith et al., 2014). In other cases, such as the UK’s pioneering 2005 Building Regulation requirement for gas and oil boilers to be condensing, the change has been almost invisible outside the relevant industry sector (Elwell et al., 2015). Public pressure on the broad direction of policy is clearly helpful, but many detailed and low-visibility policy changes driven by institutional changes are also needed. The broader understanding of the role of socio-technical change in transitions that has developed significantly since 1989 should help analysts be more open to these factors in future.

This remains a critical uncertainty for the next 30 years, in which the changes in the way energy is supplied and used will need to be more radical than in the last 30 years, if the net zero target is to be reached. As well as technical change, this will require social innovation, social change and political leadership including at local level (Martiskainen, 2017). However, there is cause for optimism. For example, community energy which aims to put people at the heart of the energy system was unknown 20 years ago. Over 5000 community energy groups in UK have now built local projects on renewable electricity generation, energy efficiency, heat networks, energy supply arrangements and energy storage; all are either wholly owned and/or controlled by communities or through partnership with commercial or public sector partners (Community Energy England, 2020). There are also signs of political movement at higher levels; for example, Scotland has the goal of reaching 2 GW of renewable energy capacity in local ownership by 2030 (EST, 2020). Combined with the political pressure ranging from new movements such as school strikes and Extinction Rebellion to the increasing number of pension funds that publically say that they do not want to invest in fossil fuels (Bergman, 2018), there is hope for the future. What can be achieved remains uncertain, but the key lesson from the last 30 years is that change is possible.
